# Spontaneous Ovarian Hyperstimulation Syndrome Associated With Primary Hypothyroidism

**DOI:** 10.7759/cureus.33247

**Published:** 2023-01-02

**Authors:** Jawharah A Alzebidi, Khairiah Almushri, Rehab Elmoheen

**Affiliations:** 1 Reproductive Endocrinology and Infertility Consultant, Alqunfudah General Hospital, Al Qunfudhah, SAU; 2 Pathology and Histopathology, Alqunfudah General Hospital, Al Qunfudhah, SAU; 3 Obstetrics and Gynaecology, Ain Shams University, Tanta, EGY

**Keywords:** primary hypothyroidism, hypothyrodism, thyroxine, thyroid-stimulating hormone, spontaneous ovarian hyperstimulation syndrome

## Abstract

Ovarian hyperstimulation syndrome is a rare condition in pregnant women. Most cases are associated with the use of ovulation induction and stimulation medications. Some studies have reported cases of this condition in non-pregnant women or women undergoing ovulation therapy. In this case report, we report the case of a 27-year-old pregnant Saudi woman presenting with a picture of severe spontaneous ovarian hyperstimulation syndrome secondary to severe undiagnosed hypothyroidism. Treatment with Eltroxin (thyroxine) led to complete improvement and regression of ovarian enlargement after empirical titrating thyroxin replacement therapy, which proved the presence of this causation. The diagnosis was confirmed by laboratory and imaging findings, which helped prompt management and prevented complications of unneeded surgical intervention.

## Introduction

Spontaneous ovarian hyperstimulation syndrome is an uncommon condition in pregnancies conceived naturally. Ovarian hyperstimulation syndrome rarely occurs in the absence of exogenous gonadotropins. Few reported cases have been documented in the literature [1-8]. Ovarian hyperstimulation syndrome is common in patients undergoing ovulation induction therapy with a rate of 0.2%-1%. Spontaneous occurrence is rare in patients do not who use ovulation induction therapies [9]. Symptoms associated with this condition include bilateral symmetric enlargement of ovaries with cysts of varying sizes, associated with pericardial effusion, pleural effusion, or ascites in severe cases. Van Wyk and Grumbach gave the first description of combined multicystic ovaries, precocious puberty, and juvenile hypothyroidism in 1960, and since then, several cases of this condition have been reported in adolescents or prepubertal girls [10-13]. Very few cases have been observed in adults, both gestational and non-gestational [1-8]. Studies have shown a likelihood of spontaneous ovarian hyperstimulation syndrome occurring at 3-8 weeks of gestation [14,15]. In this case report, we describe the occurrence of spontaneous ovarian hyperstimulation syndrome in a 27-year-old pregnant Saudi woman with a history of normal pregnancy without the use of induction of ovulation medication of exogenous herbal medication, which ended with spontaneous vaginal delivery of a normal baby at term gestation and without complications. Very few cases have been reported in the literature [16]. This case illustrates the role and effectiveness of levothyroxine empirically with gradual dose reduction in the treatment of a patient with spontaneous ovarian hyperstimulation with hypothyroidism.

## Case presentation

The patient is a 27-year-old Saudi woman who presented to our facility referred case from a private hospital for possible surgical intervention as an ovarian tumor with abdominal distention and nausea. She is gravida 2, Para 1 (G2P1+0) at 10 weeks gestation and has no abdominal pain, vomiting, or per vaginal bleeding. Not known to have any chronic medical diseases and is not on regular medications with no previous history of any surgical intervention. The patient also had no history of medications or herbal use for induction of ovulation. She was admitted for further investigation and management. Blood investigations were within normal ranges except for high thyroid-stimulating hormone (TSH) of 123 µIU/ml (normal value is 0.35 - 4.0 µIU/ml) and low level of free thyroxine (T4), at 0.21 (normal value is 0.8 - 1.8 ng/dl), indicating hypothyroidism which was undiagnosed previously in her case. Her pregnancy was confirmed biochemically by hormonal assay and radiologically by ultrasound examination.

The transvaginal ultrasound showed large bilateral ovarian cysts with mild fluid collection (ascites) and thin septations (Video 1).

**Video 1:** Transvaginal ultrasound shows multiple ovarian cysts

**Figure 1:**
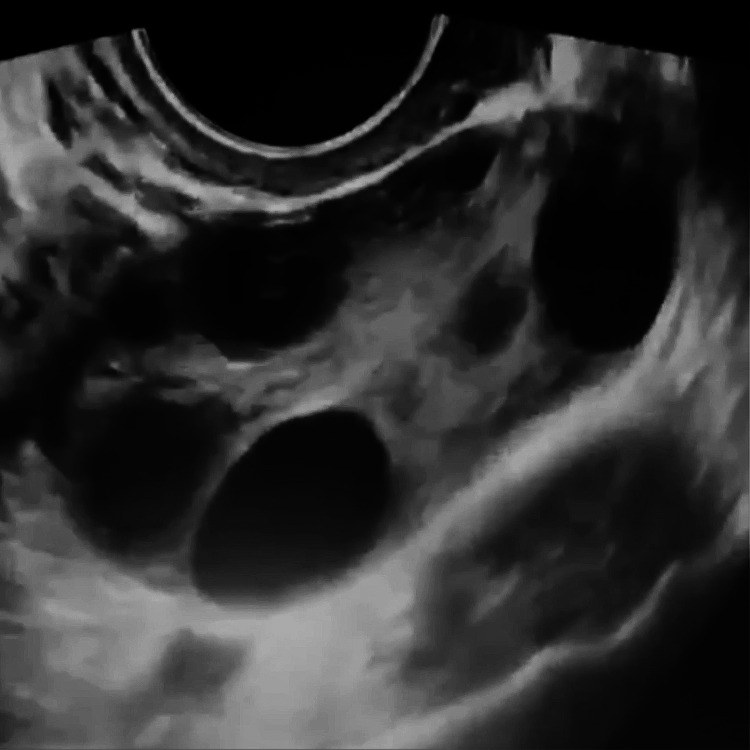
Transvaginal ultrasound shows an ovary with multiple cysts

The right ovary measures 10 x 9.7 x 11.7 cm (anteroposterior plane (AP) x transverse plane (TRANS) x craniocaudal plane (CC)) and has a volume of 323.6 ccs. Measurement of the left ovary was at 7.5 x 10.5 x 10.3 cms (AP) x (TRANS) x (CC), with a volume of 395.4 ccs. Picture suggestive of ovarian hyperstimulation syndrome (Figure 1).

The patient was given the possibility of ovarian malignancies in another facility. Therefore, CA-125 was done, and the results were normal. Additional hormonal investigations due to abnormal thyroid function tests were performed. The test results (Table 1) showed high estradiol (E2) at a value of 758.93 Picogram per milliliter (Pg/ml) (normal, < 56 pg/ml). The follicle-stimulating hormone (FSH) was slightly raised at a level of 11.92 milli-international units per ml (mIU/ml) (normal 2.5-10.2 mIU/ml). The luteinizing hormone (LH) was normal, with a value of less than 3.0 International Units per liter (IU/L) (normal < 5.0).

**Table 1:** Blood investigations of the case FSH: Follicle-stimulating hormone, LH: Luteinizing hormone, E2: Estradiol, HCG: Human chorionic gonadotropin TSH: Thyroid-stimulating hormone, T4: Free thyroxin, CA-125: Cancer antigen 125

BLOOD INVESTIGATION	RESULT	NORMAL RANGE
FSH	11.92	2.5 - 10.2 mIU/ml
LH	<3	<5 IU/l
E2	758.93	<56 pg/ml
HCG	23150	< 6.4 mIU/ml
TSH	123	0.35-4.0 mIU/ml
T4	0.21	0.8-1.8 ng/dl
CA-125	11	<35 IU/ml

A spontaneous ovarian hyperstimulation syndrome diagnosis was made based on radiological and clinical findings. The patient started on levothyroxine at a daily dose of 300 micrograms (mcg) for two days. The dose was gradually reduced to 100 mcg and continued until delivery. This regimen leads to a complete reduction of ovarian enlargement and elimination of ascites, abdominal distention, and respiratory symptoms by three months duration postpartum.

## Discussion

De Leener et al. reported spontaneous ovarian hyperstimulation syndrome could occur in both pregnant and non-pregnant women [17]. Ovarian hyperstimulation syndrome could be classified into three types based on FSH receptor mutation and clinical presentation. Type 1 is due to mutation of the FSH receptor and may result in recurrent hyperstimulation syndrome. Type II occurs secondary to high levels of human chorionic gonadotropin (hCG) as in multiple gestation and hydatiform mole and is common. Type III is associated with hypothyroidism which is rare [17].

Controlled ovarian stimulation is a risk factor for ovarian hyperstimulation syndrome. In rare cases, it may occur as a spontaneous event in pregnancy.

Risks for developing ovarian hyperstimulation syndrome include asthenic habitus, polycystic ovarian syndrome, luteal supplementation of hCG, multiple follicles, high levels of estradiol in the serum, and GnRH agonist protocols [18].

Some studies have reported spontaneous ovarian hyperstimulation syndrome between the 8^th^ and 14^th^ weeks of pregnancy as a rare occurrence. Cases have also been reported with follicle-stimulating (FSH) producing pituitary adenoma. There have also been reports of this syndrome in rare cases of increased hCG production, which is typical in multiple pregnancies, hydatidiform mole, polycystic ovary disease, and increased thyroid-stimulating hormone levels in hypothyroidism [19].

More studies are needed to know the etiology of spontaneous ovarian hyperstimulation syndrome. However, very few cases of ovarian hyperstimulation syndrome have been documented with pregnancy.

The pathophysiology of ovarian hyperstimulation syndrome in hypothyroid conditions is still unclear and needs further studies. Rotmensch and Scommegna explained the estriol formation via the 16-hydroxylation pathway rather than the 2-hydroxylation pathway typical of hypothyroid patients [20]. Due to a reduction in feedback regulation, a high release of gonadotropin caused by substituting estradiol with estriol would cause high ovarian stimulation [2].

Cardoso illustrated large bilateral ovarian cyst regression in a patient with the hypothyroid condition after thyroid hormone replacement therapy, indicating a relationship between spontaneous ovarian hyperstimulation syndrome and primary hypothyroidism [2]. A case report by Nappi et al. showed the effectiveness of thyroid replacement therapy and fluid administration in a previously untreated hypothyroid patient with spontaneous ovarian hyperstimulation syndrome [20].

The patient, in our case, had very high TSH levels with low levels of free thyroxine, which indicate hypothyroidism. 

Ovarian hyperstimulation syndrome (OHSS) has varying clinical symptoms, which depend on its severity. Symptoms may be classified as mild, moderate, severe, and critical. In mild OHSS, ovarian enlargement is ‘uncomplicated,’ and with multiple cysts, ascites characterizes moderate conditions, while severe forms are associated with pericardial or pleural effusion, thrombosis, hemoconcentration, and oliguria [21]. The critical form required intensive care unit (ICU) admission with possible termination of pregnancy. 

Symptoms are mostly due to fluid accumulation in the extravascular space as well as in the third space due to the high permeability of the membranes. The resulting complications may be electrolyte imbalance, hypo-albuminemia, oliguria, decreased renal perfusion, and pericardial and pleural effusions, leading to respiratory failure, liver, and renal failure, as well as hemoconcentration and thrombosis formation [22].

Imaging in MRI, CT, and US may be similar. Findings may include pleural effusion, ascites, and cysts alongside bilateral enlarged ovaries. OHSS may be complicated by ovarian torsion [23]. Our patient has moderate ovarian hyperstimulation syndrome.

The radiological finding can give the wrong impression of an ovarian tumor which might lead to unnecessary intervention for the patient, so careful assessment and a high index of suspicions of other differential diagnoses should be considered.

Mild or moderate OHSS may be treated with conservative management. Severe conditions are usually characterized by the depletion of body fluids which may be replaced with normal saline or ringer lactate. It is important that the clinician determines the underlying cause and treat it accordingly. In our case, the patient had OHSS with hypothyroidism and was treated with thyroxine.

## Conclusions

This case study has illustrated the occurrence of spontaneous ovarian hyperstimulation. Patients with similar conditions should be tested for hypothyroidism, as this has the potential to cause spontaneous OHSS. Early diagnosis can help in prompt management and prevent unnecessary surgical intervention and complications.
